# Oleate lipase activity in *Gardnerella vaginalis *and reconsideration of existing biotype schemes

**DOI:** 10.1186/1471-2180-9-78

**Published:** 2009-04-22

**Authors:** Bernard J Moncla, Kara M Pryke

**Affiliations:** 1 Department of Obstetrics, Gynecology and Reproductive Sciences, University of Pittsburgh, Pittsburgh, PA 15213-3180, USA; 2 Magee-Womens Research Institute, University of Pittsburgh, Pittsburgh, PA 15213-3180, USA

## Abstract

**Background:**

*Gardnerella vaginalis *is a facultative gram positive organism that requires subculture every 1–2 days to maintain viability. It has been linked with bacterial vaginosis (BV), a syndrome that has been associated with increased risk for preterm delivery, pelvic inflammatory disease and HIV acquisition. About 10% of the *G*. *vaginalis *isolates have been reported to produce sialidase, but there have not been any studies relating sialidase production and biotype. Sialidase activity is dramatically increased in the vaginal fluid of women with BV and bacterial sialidases have been shown to increase the infectivity of HIV *in vitro*. There are 8 different biotypes of *G. vaginalis*. Biotypes 1–4 produce lipase and were reported to be associated with BV and the association of these biotypes with BV is under dispute. Other studies have demonstrated that *G. vaginalis *biotype 1 can stimulate HIV-1 production. Because of the discrepancies in the literature we compared the methods used to biotype *G. vaginalis *and investigated the relationship of biotype and sialidase production.

**Results:**

A new medium for maintenance of *Gardnerella vaginalis *which allows survival for longer than one week is described. Some isolates only grew well under anaerobic conditions. Sialidase producing isolates were observed in 5 of the 6 biotypes tested. Using 4-methylumbelliferyl-oleate to determine lipase activity, instead of egg yolk agar, resulted in erroneous biotypes and does not provide reliable results.

**Conclusion:**

Previous studies associating *G. vaginalis *biotype with bacterial vaginosis were methodologically flawed, suggesting there is not an association of *G. vaginalis *biotypes and bacterial vaginosis. Sialidase activity was observed in 5 of the 8 biotypes.

## Background

Bacterial vaginosis (BV) is one of the most common reasons for women to seek medical attention; the underlying cause of BV is controversial. Women with BV are at higher risk for preterm delivery, pelvic inflammatory disease (PID) and acquisition of HIV [1-5]. Several reports have demonstrated that women with BV have elevated levels of sialidase activity [6-9], although the source has not been demonstrated. Studies have demonstrated that treatment of HIV-1 or lymphocytes with bacterial sialidase increases the infectivity of the virus [10,11]. A number of different bacteria have been associated with BV, including *Gardnerella vaginalis *[12-14]. *G. vaginalis *can be isolated from women without any symptoms and recovered from sites which are usually sterile [15,16]. Studies have also shown *G. vaginalis *biotype 1 fractions are capable of stimulating HIV-1 production [17]. *G. vaginalis *is a fastidious organism requiring subculture to fresh media every two days. Isolates identified as *G. vaginalis *may be further characterized by β-galactosidase and lipase activity and hippurate hydrolysis resulting in 8 biotypes [18]. According to one study biotypes 1–4 produce lipase and in a longitudinal study were significantly associated with BV. After successful treatment, the predominant *G. vaginalis *biotypes shifted to non-lipase producing types 5–8 [6]. Other studies did not find a relationship between BV and biotype or genotype [15]. Piot et al. [18] defined biotypes using egg yolk agar (EY) to test for lipase while Briseldon and Hillier [6] used 4-methylumbelliferyl-oleate (MUO). Since the use of MUO had not been validated, we compared the results of lipase detection using egg yolk to those obtained with MUO. Because *G. vaginalis *sialidase could play an important role in both BV and HIV infection we also tested our strains for sialidase activity.

## Methods

### *Gardnerella vaginalis *agar (GVA)

Most of our work is performed with strains with a low number of passages; we therefore devised a medium for *G. vaginalis *that facilitated our work by not requiring frequent subculture to fresh medium. GVA was prepared by dissolving: Brain Heart Infusion 37 g, Bacto-Tryptone 5 g, yeast extract 1 g, soluble starch 1 g, KH_2_PO_4 _6.8 g, and L-cysteine HCl 0.3 g in 1 liter of distilled water. The pH was then adjusted to 7.2 with sodium hydroxide and Bacto-agar added to a final concentration 1.5% and the medium sterilized by autoclaving. The medium was cooled and dispensed to 100 mm plastic Petri plates, then air dried for 30 min and stored at 4°C. To analyze the survival on GVA the *G. vaginalis *isolates were cultured from blood agar plates (BAP) onto GVA plates on the first day. Subcultures were made from the first day GVA plates onto a fresh BAP and GVA daily for one week or until the subcultures failed to grow.

### Bacteria

The reference strain of *G. vaginalis*, ATCC 14018, was obtained from the American Type Culture Collection. Human vaginal isolates of *G. vaginalis *were kindly provided by Lorna Rabe, (Magee Womens Research Institute, Pittsburgh PA). Initial identifications were based on colony morphology, Gram stain, lack of catalase activity and hemolysis on human bilayer tween medium (HBT) but not sheep blood agar. Isolates were stored frozen at -80°C until needed, then revived and maintained on Columbia blood agar containing 5% sheep's blood PML Microbiologicals (PML Microbiologicals, Wilsonville, OR.).

### Biotyping

β-galactosidase, lipase activity and hippurate hydrolysis were performed as described previously [18]. Egg yolk agar plates for lipase reactions were obtained from PML Microbiologicals (PML Microbiologicals, Wilsonville, OR.) were inoculated and examined daily for 7 days. The presence of an oily sheen on and surrounding the bacterial growth was interpreted as a positive result for lipase activity. *Staphylococcus aureus *and *Pseudomonas aeruginosa *were used as positive controls, *Lactobacillus crispatus *was used as a negative control.

### Lipase activity using 4-methylumbelliferyl-oleate

Lipase activity was also determined as described by Briselden and Hillier [6], using 4-methylumbelliferyl-oleate (Fluka 75164, from the Sigma Aldrich Chemical Co., St. Louis, MO.) using a spot test as previously described [6,19]. Briefly, MUO was dissolved in absolute ethanol to a concentration of 4 mg/ml and used to soak a 60 by 6 mm (approximate) Whatman #2 filter paper strip (Whatman, Inc., Clifton, N.J.). After air drying, strips were moistened with buffer, phosphate buffered saline or ACES both pH 7.0 containing 22 mM *N*-octyl-β-D-glucopyranoside, and 12 mM CaCl_2_. Alternatively, the MUO was suspended in water and mixed with equal parts of 2 fold concentrated buffer as described above. The strips were inoculated with a loopful of bacteria, incubated at 35°C, and read under a long-wavelength (365 nm), hand-held mineral lamp as described previously [19].

### Sialidase activity using 2'(4-methylumbelliferyl) – α-D-N-acetylneuraminic acid

Sialidase activity was determined as described previously by Moncla et al. [19] using 4-methylumbelliferyl- α-D-N-acetylneuraminic acid as substrate (Sigma M8639, from the Sigma Aldrich Chemical Co., St. Louis, MO.). Stock solutions of the substrate were prepared by dissolving 1 mg in 6.6 ml distilled water, dispensing into 180 μl volumes and storing at -20°C. The stock solutions were thawed and 20 μl 1.0 M sodium acetate buffer, pH 4.8 added and mixed in. The resulting solutions were used in a filter paper strip assay as described above for the 4-methylumbelliferyl-oleate lipase assay.

## Results

All strains survived for 7 days on GVA (Table 1) and by week three about one third of the strains were viable. We did not find any isolates representing biotypes 6 and 8. Several isolates grew little if at all on the GVA or blood agar aerobically; however, good growth was observed for all isolates when cultured anaerobically. On EY (see below), 7 of the isolates failed to grow in air plus 6% CO_2_ but did grow well anaerobically (Table 1). Strains grown on GVA gave identical biochemical reactions for β-galactosidase, sialidase and hippurate hydrolysis as when grown on BAP.

**Table 1:** Characteristics of *Gardnerella vaginalis *isolates on GVA: Biotypes, strain designations, growth and enzymatic activities

Isolate designation (Biotype)^A^		48 hr growth GVA plates	Viability at 7 days On GVA plates^B^	Aerobic Growth on EY	Lipase Egg yolk^C^	Lipase with 4-MUO	Sialidase
0284	(1)	+	+	+	+	-	+
0287	(1)	+	+	+	+	-	+
0828E	(1)	+	+	+	+	-	-
0377	(1)	+	+	+	+	-	-
ATCC 14018	(1)	+	+	+	+	-	-
C31g 7571–2	(1)	+	+	+	+	-	-
ITP	(1)	+	+	+	+	-	-
PID 0286E	(1)	+	+	+	+	+	+
UPS 1388E	(1)	+	+	-	+	+	-
UPS1492	(1)	+	+	+	+	-	+
551	(2)	+	+	+	+	-	-
UPS1476	(2)	+	+	+	+	+	-
HPTN							
208000837	(2)	+	+	+	+	-	+
UC781 64/20- LITTLE	(2)	+	+	+	+	+	+
UC781 64/20- BIG	(2)	+	+	+	+	+	+
UP548	(2)	+	+	-	+	-	+
UPS 754	(2)	+	+	+	+	-	-
055/15	(3)	+	+	+	+	+	-
UP537 E	(3)	+	+	+	+	-	-
RING52576	(4)	+	+	+	+	+	-
HPTN 213000700	(4)	+	+	+	+	-	+
UPS 1400E	(4)	-/scant^D^	+	+	+	-	-
UPS 1398Ea	(5)	+	+	-	-	-	+
UPS 1483E	(5)	-	+	-	-	+	-
UPS 1500E	(5)	+	+	-	-	-	-
061/19 V5	(7)	+	+	+	-	+	+
HPTN008 000750	(7)	-	+	-	-	+	-
Up 582	(7)	+	+	+	-	+	-
UPS 1401 E	(7)	+	+	+	-	+	+
UPS 2050E	(7)	+	+	+	-	-	-
UPS1475	(7)	+	+	-	-	+	-

^**A**^The biotype of each isolate is given in parentheses.

^**B**^To accommodate those isolates demonstrating better growth anaerobically all strains were incubated in anaerobe jars. Aerobic organisms survived equally well under either incubation condition. Cultures were considered viable after 7 days if they were successfully subcultured to fresh GVA and blood agar plates.

^**C**^All strains were tested for lipase production on egg yolk agar under aerobic and anaerobic conditions; strains demonstrating growth aerobically yield identical lipase reaction when grown anaerobically. When strains did not grow aerobically on egg yolk plates the reactions indicated are taken from anaerobic incubation. Lipase reactions using 4-methylumbelliferone-oleate are given in parentheses.

^**D**^Some organisms demonstrated poor growth when incubated in air plus 6% CO_2_, these organisms all had excellent growth on GVA plates after anaerobic incubation.

Lipase activity was detected in 21 of 31 strains tested (68%) using egg yolk agar but using the MUO spot test only 12 of 31 strains tested positive (39%) (Table 1). To assess the performance of the MUO based lipase test, the egg yolk reactions were used as the true values in the statistical comparison as described previously by Moncla et al. [20]. The values obtained for the MUO test were: sensitivity (32%), specificity (33%), positive predictive value (54%) and negative predictive value (17%). The alternate method for lipase detection (see above, mixing equal volumes of buffer and liquid substrate) demonstrated a lack of reproducibility and the results from these assays are not presented.

Sialidase was detected in one or more strains of biotypes 1, 2, 4, 5 and 7 but not in strains from biotype 3, (Table 1). Biotypes 6 and 8 were not found among the strains examined. Most of the strains studied were biotype 1 (32.2%), followed by biotypes 2, 7, 4, 5, and 3 (22.5%, 19.5%, 9.6%, 9.6%, 6.5% respectively). Overall, the 39% of the strains tested demonstrated sialidase activity.

## Discussion

GVA provides an inexpensive alternative to the long term cultivation of *G. vaginalis*, compared to blood agar or HBT on which the organism requires passage every day or two. Some isolates survived for up to three weeks on the GVA. HBT is an important medium commonly used to test the hemolysis characteristics and to maintain the organism; however, it is too expensive for long term passage of the organism.

Sialidase activity is an important feature in bacterial vaginosis. Accordingly, we concurrently tested our strains for the enzyme. Previous reports had noted the enzyme in 10% of the isolates [21]. We observed higher rates among our isolates 39% (table 1). About one third of our isolates were biotype 1, of which 40% of the isolates were positive. The presence of sialidase was detected in strains from all biotypes tested except biotype 3; however, we only had three isolates identified as biotype 3. We did not identify any isolates as biotypes 6 or 8.

The sialidase activity associated with BV is most likely from bacterial sources, although the specific organisms have not been identified. We report a higher incidence of the enzyme than reported by others [21] the significance is difficult to access since we only examined 31 strains. Because they are found in low frequency we did not test biotype 6 or 8. Other bacteria associated with BV have a much greater percentage of isolates that produce sialidase for example; all isolates of *Prevotella bivia *produced the enzyme [19]. While *P. bivia *isolates all produce sialidase, only about 3% of the activity is released into the medium; however, we do observe surprisingly large amounts of sialidase in the culture supernatants of sialidase producing strains of *G vaginalis *(Moncla unpublished observations).

Because of the relationship between *G. vaginalis *biotype and the stimulation of HIV replication [12,17]; we attempted to biotype our strains using the MUO method of Briselden and Hillier. *G. vaginalis *ATCC 14018 is biotype 1; however because of a negative lipase reaction we consistently identified it as biotype 6. As the MUO method of Briselden and Hillier had not been validated we compared the results of lipase detection using egg yolk agar to those obtained with MUO. The choice of lipase substrate had a dramatic effect on the biotype. For example, using EY lipase results there are 10 strains listed in Table 1 as biotype 1; however, using MUO as the substrate, only 2 of the 10 isolates demonstrated lipase activity. The 8 strains that were negative using MUO would have been incorrectly identified as biotype 6. Overall 20 of the tested isolates would have yielded a different biotype depending on the method used for testing lipase activity. The MUO method had poor sensitivity and specificity when calculated as described previously [20]. Using the EY data for biotyping as presented in Table 1, we observed a distribution of biotypes that is roughly similar to that reported by Piot et al.

Lipases are specialized esterases; therefore, which synthetic substrates, if any, are appropriate is controversial. Studies suggest synthetic substrates such as MUO detect non-specific esterase activity [22-27]. Our data would support this concept. When other 4-methylumbelliferyl fatty acids were used, we observed all strains give a positive test results with MU-heptonate but none with 4-methylumbelliferyl palmitic acid, indicating the assays are measuring esterase activity [28,29]. These data would tend to negate the observations of others regarding the correlation of *G. vaginalis *biotype with BV. Briselden and Hillier's observation of a reduction of lipase producing biotype 1–4 after successful treatment could be reinterpreted as an association of non-specific esterase activity in *G. vaginalis *with BV [6]. Our results demonstrate the importance of lipase activity in the typing of *G. vaginalis *and that lipase activity should be tested using EY plates or other lipase assay methods such as titration. Further, our work suggests the reports of biotypes using the MUO or other 4-methyumbelliferone substrates in lipase spot tests are not accurate. Other differences exist in the methodologies reported, Piot et al. and Briselden and Hillier grew cultures anaerobically while the other groups mentioned grew organisms aerobically with enriched CO_2_. Lipase reactions on EY often take up to 7 or more days, and all these groups used only 3 days or less for reactions. Our observations suggest all isolates should be cultured anaerobically, and EY plates should be incubated for 7 days before they can be interpreted as lipase negative.

In summary a medium was described that allows survival of *G. vaginalis *isolates for at least one week and longer in some cases. Sialidase activity was observed in 40% of the strains tested but was not restricted to any particular biotypes. The synthetic lipase substrate 4-methylumbelliferyl-oleate did not reliably detect lipase activity compared to egg yolk plates.

## Conclusion

Our data suggests the relationship of BV and *G. vaginalis *biotype should be reexamined, since our study demonstrates that 4-methylumbelliferyl-oleate and other 4-methylumbelliferyl- derivatives should not be used for the detection of lipase activity as a tool for bacterial identifications. The *Gardnerella vaginalis *agar allows extended viability of the cultures, therefore the time and costs of frequent subculture is greatly reduced. We cannot rule out an association of *G. vaginalis*, sialidase, BV and increases in HIV acquisition rates among women with BV.

## Abbreviations

BV: bacterial vaginosis; EY: egg yolk agar; GVA: *Gardnerella vaginalis *agar; HBT: bilayer tween medium; MU: 4-methylumbelliferyl; MUO: 4-methylumbelliferyl-oleate; PID: pelvic inflammatory disease.

## Authors' contributions

BJM conceived the study, directed the experimental designs and carried out assays. He prepared the draft of manuscript and final versions of the manuscript. KMP contributed to the study conception and the experimental designs. She performed assays and microbiological aspects of study. KMP help to prepare and edit the manuscript. Both authors have read and approved the manuscript.

## References

[B1] Leitich H Bodner-Adler B Brunbauer M Kaider A Egarter C Husslein P Bacterial vaginosis as a risk factor for preterm delivery: a meta-analysis Am J Obstet Gynecol 2003 189 1 139 147 10.1067/mob.2003.339 12861153

[B2] Marrazzo JM A persistent(ly) enigmatic ecological mystery: bacterial vaginosis J Infect Dis 2006 193 11 1475 1477 10.1086/503783 16652273

[B3] Marrazzo JM Wiesenfeld HC Murray PJ Busse B Meyn L Krohn M Hillier SL Risk factors for cervicitis among women with bacterial vaginosis J Infect Dis 2006 193 5 617 624 10.1086/500149 16453256

[B4] Taha TE Dallabetta GA Hoover DR Chiphangwi JD Mtimavalye LA Liomba GN Kumwenda NI Miotti PG Trends of HIV-1 and sexually transmitted diseases among pregnant and postpartum women in urban Malawi Aids 1998 12 2 197 203 10.1097/00002030-199802000-00010 9468369

[B5] Taha TE Hoover DR Dallabetta GA Kumwenda NI Mtimavalye LA Yang LP Liomba GN Broadhead RL Chiphangwi JD Miotti PG Bacterial vaginosis and disturbances of vaginal flora: association with increased acquisition of HIV Aids 1998 12 13 1699 1706 10.1097/00002030-199813000-00019 9764791

[B6] Briselden AM Hillier SL Longitudinal study of the biotypes of Gardnerella vaginalis J Clin Microbiol 1990 28 12 2761 2764 2280007 10.1128/jcm.28.12.2761-2764.1990 PMC268269

[B7] Cauci S Driussi S Monte R Lanzafame P Pitzus E Quadrifoglio F Immunoglobulin A response against Gardnerella vaginalis hemolysin and sialidase activity in bacterial vaginosis Am J Obstet Gynecol 1998 178 3 511 515 10.1016/S0002-9378(98)70430-2 9539518

[B8] McGregor JA French JI Jones W Milligan K McKinney PJ Patterson E Parker R Bacterial vaginosis is associated with prematurity and vaginal fluid mucinase and sialidase: results of a controlled trial of topical clindamycin cream Am J Obstet Gynecol 1994 170 4 1048 1059 discussion 1059–1060. 8166188 10.1016/s0002-9378(94)70098-2

[B9] Wiggins R Crowley T Horner PJ Soothill PW Millar MR Corfield AP Use of 5-bromo-4-chloro-3-indolyl-alpha-D-N-acetylneuraminic acid in a novel spot test To identify sialidase activity in vaginal swabs from women with bacterial vaginosis J Clin Microbiol 2000 38 8 3096 3097 10921986 10.1128/jcm.38.8.3096-3097.2000 PMC87196

[B10] Hu H Shioda T Moriya C Xin X Hasan MK Miyake K Shimada T Nagai Y Infectivities of human and other primate lentiviruses are activated by desialylation of the virion surface Journal of virology 1996 70 11 7462 7470 8892864 10.1128/jvi.70.11.7462-7470.1996 PMC190813

[B11] Stamatos NM Gomatos PJ Cox J Fowler A Dow N Wohlhieter JA Cross AS Desialylation of peripheral blood mononuclear cells promotes growth of HIV-1 Virology 1997 228 2 123 131 10.1006/viro.1996.8373 9123818

[B12] Fleming DT Wasserheit JN From epidemiological synergy to public health policy and practice: the contribution of other sexually transmitted diseases to sexual transmission of HIV infection Sex Transm Infect 1999 75 1 3 17 10448335 10.1136/sti.75.1.3 PMC1758168

[B13] Martin HL Richardson BA Nyange PM Lavreys L Hillier SL Chohan B Mandaliya K Ndinya-Achola JO Bwayo J Kreiss J Vaginal lactobacilli, microbial flora, and risk of human immunodeficiency virus type 1 and sexually transmitted disease acquisition J Infect Dis 1999 180 6 1863 1868 10.1086/315127 10558942

[B14] van Esbroeck M Vandamme P Falsen E Vancanneyt M Moore E Pot B Gavini F Kersters K Goossens H Polyphasic approach to the classification and identification of Gardnerella vaginalis and unidentified Gardnerella vaginalis-like coryneforms present in bacterial vaginosis Int J Syst Bacteriol 1996 46 3 675 682 8782675 10.1099/00207713-46-3-675

[B15] Aroutcheva AA Simoes JA Behbakht K Faro S Gardnerella vaginalis isolated from patients with bacterial vaginosis and from patients with healthy vaginal ecosystems Clin Infect Dis 2001 33 7 1022 1027 10.1086/323030 11528575

[B16] Lien EA Hillier SL Evaluation of the enhanced rapid identification method for Gardnerella vaginalis J Clin Microbiol 1989 27 3 566 567 2785533 10.1128/jcm.27.3.566-567.1989 PMC267361

[B17] Simoes JA Hashemi FB Aroutcheva AA Heimler I Spear GT Shott S Faro S Human immunodeficiency virus type 1 stimulatory activity by Gardnerella vaginalis: relationship to biotypes and other pathogenic characteristics J Infect Dis 2001 184 1 22 27 10.1086/321002 11398105

[B18] Piot P Van Dyck E Peeters M Hale J Totten PA Holmes KK Biotypes of Gardnerella vaginalis J Clin Microbiol 1984 20 4 677 679 6333436 10.1128/jcm.20.4.677-679.1984 PMC271409

[B19] Moncla BJ Braham P Hillier SL Sialidase (neuraminidase) activity among gram-negative anaerobic and capnophilic bacteria J Clin Microbiol 1990 28 3 422 425 2108991 10.1128/jcm.28.3.422-425.1990 PMC269635

[B20] Moncla BJ Braham PH Persson GR Page RC Weinberg A Direct detection of Porphyromonas gingivalis in Macaca fascicularis dental plaque samples using an oligonucleotide probe J Periodontol 1994 65 5 398 403 8046554 10.1902/jop.1994.65.5.398

[B21] von Nicolai H Hammann R Salehnia S Zilliken F A newly discovered sialidase from Gardnerella vaginalis Zentralbl Bakteriol Mikrobiol Hyg [A] 1984 258 1 20 26 6335332 10.1016/s0176-6724(84)80003-6

[B22] Arpigny JL Jaeger KE Bacterial lipolytic enzymes: classification and properties Biochem J 1999 343 1 177 183 10493927 10.1042/0264-6021:3430177 PMC1220539

[B23] Burdette RA Quinn DM Interfacial reaction dynamics and acyl-enzyme mechanism for lipoprotein lipase-catalyzed hydrolysis of lipid p-nitrophenyl esters J Biol Chem 1986 261 26 12016 12021 3745178

[B24] Hendrickson HS Fluorescence-based assays of lipases, phospholipases, and other lipolytic enzymes Anal Biochem 1994 219 1 1 8 10.1006/abio.1994.1223 8059934

[B25] Jaeger K-E Ransac S Dijkstra BW Colson C Heuvel M Misset O Bacterial lipases FEMS Microbiology Reviews 1994 15 1 29 63 10.1111/j.1574-6976.1994.tb00121.x 7946464

[B26] Miles RJ Siu EL Carrington C Richardson AC Smith BV Price RG The detection of lipase activity in bacteria using novel chromogenic substrates FEMS Microbiol Lett 1992 69 3 283 287 10.1111/j.1574-6968.1992.tb05167.x 1555763

[B27] Thomson CA Delaquis PJ Mazza G Detection and Measurement of Microbial Lipase Activity: A Review Critical Reviews in Food Science and Nutrition 1999 39 2 165 187 10.1080/10408399908500492 10198753

[B28] Beisson F Tiss A Rivière C Verger R Methods for lipase detection and assay: a critical review European Journal of Lipid Science and Technology 2000 102 2 133 153 10.1002/(SICI)1438-9312(200002)102:2<133::AID-EJLT133>3.0.CO;2-X

[B29] Bornscheuer UT Microbial carboxyl esterases: classification, properties and application in biocatalysis FEMS Microbiol Rev 2002 26 1 73 81 10.1111/j.1574-6976.2002.tb00599.x 12007643

